# Prognostic value of prostate volume in non-muscle invasive bladder cancer

**DOI:** 10.1038/s41598-021-98045-1

**Published:** 2021-09-22

**Authors:** Won Sik Ham, Jee Soo Park, Won Sik Jang, Young Deuk Choi, Jongchan Kim

**Affiliations:** 1 Department of Urology and Urological Science Institute, Yonsei University College of Medicine, Seoul, South Korea; 2 Department of Urology, Sorokdo National Hospital, Goheung, South Korea; 3 Department of Urology, Yongin Severance Hospital, Yonsei University Health System, 363, Dongbaekjukjeon-daero, Giheung-gu, Yongin-si, Gyeonggi-do 16995, South Korea

**Keywords:** Bladder cancer, Prostatic diseases

## Abstract

There is evidence that a history of benign prostatic hyperplasia increases the incidence of bladder cancer, and treatment with 5-alpha reductase inhibitor or androgen deprivation therapy reduces recurrence of non-muscle invasive bladder cancer. We aimed to evaluate whether prostate volume affects its prognosis. We reviewed medical records of men who underwent transurethral resection of bladder tumor due to non-muscle invasive bladder cancer from January 2012 to December 2017. Patients were divided into two groups based on prostate volume measured by computed tomography (group 1: 264 patients with ≤ 30 mL, group 2: 124 patients with > 30 mL). Propensity score matching analysis was used for adjust selection bias, and then assessed recurrence-free survival and progression-free survival. With a median follow up duration of 52 months, group 1 showed higher 5-year recurrence-free and progression-free survival (69.3% vs 47.0%, p = 0.001; 96.7% vs 87.7%, p = 0.002). Further, cox-regression analysis showed that tumor size (HR = 1.292 p < 0.001), multifocal tumor (HR = 1.993, p < 0.001), adjuvant intravesical therapy (chemotherapy: HR = 0.580, p = 0.037 and bacillus Calmette–Guérin: HR = 0.542, p = 0.004) and prostate volume (HR = 2.326, p < 0.001) were significant predictors of recurrence-free survival. Prostate volume (HR = 2.886, p = 0.014) was also associated with PFS with age (HR = 1.043, p = 0.044) and tumor grade (HR = 3.822, p = 0.013). We conclude higher prostate volume is associated with worse recurrence and progression-free survival in non-muscle invasive bladder cancer.

## Introduction

Bladder cancer is the ninth most common cancer in the world and fourth most common cancer in men^1^. Approximately 70–75% of bladder cancer cases are classified as non-muscle invasive bladder cancer (NMIBC) at the time of diagnosis^2^. Among the patients with NMIBC, 40–80% patients experience recurrence and approximately 15–40% experience progression after transurethral resection of bladder tumor (TURBT). Prior recurrence is known to be one of the risk factors for progression of NMIBC and once it has progressed, the prognosis tends to be poor^3,4^. Although radical cystectomy is needed in patients with T2 or higher grades of the disease, it is related with decrease in quality of life^5^. Hence, it is important to predict recurrence and progression of NMIBC to counsel patients.

The reported prognostic factors of NMIBC are old age, female sex, smoking history, presence of carcinoma in situ (CIS), tumor size, tumor multifocality, tumor stage, and tumor grade. The European Organization for Research and Treatment of Cancer^3,4^ and Spanish Club for Oncological Treatment^6^ group have devised progression models using some of these factors. In addition to the demographic and pathologic characteristics, intravesical therapy using bacillus Calmette–Guérin (BCG) or mitomycin-C have been found to help reduce recurrence and progression in a few patients with NMIBC^7,8^.

In addition, there have been several studies that have found other factors that are associated with the prognosis of NMIBC. Some studies have reported that presence of benign prostatic hyperplasia (BPH) is associated with increased incidence of bladder cancer^9,10^. Other studies have shown that using 5-alpha reductase inhibitors (5-ARI) in BPH or androgen deprivation therapy (ADT) in prostate cancer also reduces recurrence of bladder cancer^11,12^. BPH is treated with as it reduces prostate volume (PV)^13^. 5-ARI and ADT are inhibiting androgen receptor (AR) signaling and also known to be related to PV reduction^14^. And some studies reported AR plays roles in the development of BPH^15^. Based on these studies PV might be the indicator reflect AR and we aimed to assess if PV as an indicator associated with AR is a potential prognostic factor for NMIBC.

## Results

### Patients characteristics

Table 1 shows the patients’ clinical and pathologic characteristics of all cohort and propensity score matched cohort. The median age (69.0 vs 62.0 years, p < 0.001) and body mass index (BMI) (24.3 vs 23.7, p = 0.011) were higher in group 2 than in group 1. Tumor size was also greater in group 2 (1.8 cm vs 1.5 cm, p = 0.0.39). Additionally, high-grade tumors (60.2% vs 46.8%) and T1 stage tumors (92.6% vs 83.9%) were more frequently observed in group 2 than in group 1. There was no difference in the other variables. After propensity score matching (PSM), there was no difference in all variables between two groups.

**Table 1:** Patients characteristics of group 1 (prostate volume ≤ 30 mL) and group 2 (prostate volume > 30 mL).

Characteristics	Pre-propensity score matching			Post- propensity score matching		
	Group 1	Group 2	p-value	Group 1	Group 2	p-value
	248	108		107	107	
Age (median, IQR)	62.0 (53.5–71.0)	69.0 (62.0–76.0)	< 0.001	65.0 (57.0–74.0)	68.0 (62.0–76.0)	0.128
BMI (median, IQR)	23.7 (21.7–25.8)	24.3 (22.4–26.6)	0.011	24.2 (21.9–26.2)	24.2 (22.4–26.4)	0.242
**Smoking**			0.270			0.550
Non-smoker	64 (25.8%)	34 (31.5%)		30 (28.0%)	34 (31.8%)	
Current or former smoker	184 (74.2%)	74 (68.5%)		77 (72.0%)	73 (68.2%)	
Tumor size (median, IQR)	1.5 (1.0–2.3)	1.8 (1.0–2.8)	0.039	1.6 (1.1–2.5)	1.8 (1.0–2.8)	0.736
Multifocal tumor	119 (48.0%)	52 (48.1%)	0.977	55 (51.4%)	52 (48.1%)	0.682
**Tumor grade**			0.020			0.020
Low grade	132 (53.2%)	43 (39.8%)		51 (47.7%)	43 (40.2%)	
High grade	116 (46.8%)	65 (60.2%)		56 (52.3%)	64 (59.8%)	
**Pathologic T stage**			0.027			0.122
pTa	40 (16.1%)	8 (7.4%)		15 (14.0%)	8 (7.5%)	
pT1	208 (83.9%)	100 (92.6%)		92 (86.0%)	99 (92.5%)	
Tumor variant	14 (5.6%)	10 (9.3%)	0.211	9 (8.4%)	10 (9.3%)	0.810
CIS	10 (4.0%)	4 (3.7%)	0.883	3 (2.8%)	4 (3.7%)	> 0.999
Immediate intravesical therapy	26 (10.5%)	9 (8.3%)	0.531	8 (7.5%)	9 (8.4%)	0.800
**Adjuvant intravesical therapy**			0.201			0.537
No	138 (55.6%)	49 (45.4%)		55 (51.4%)	48 (44.9%)	
Chemotherapy	36 (14.5%)	20 (18.5%)		37 (34.6%)	39 (36.4%)	
BCG	74 (29.8%)	39 (36.1%)		15 (14.0%)	20 (18.7%)	

*IQR* interquartile range, *BMI* body mass index, *CIS* carcinoma in situ, *BCG* bacillus Calmette–Guérin.

### Recurrence-free and prognosis-free survival in the 2 groups (before PSM)

**Figure 1:**
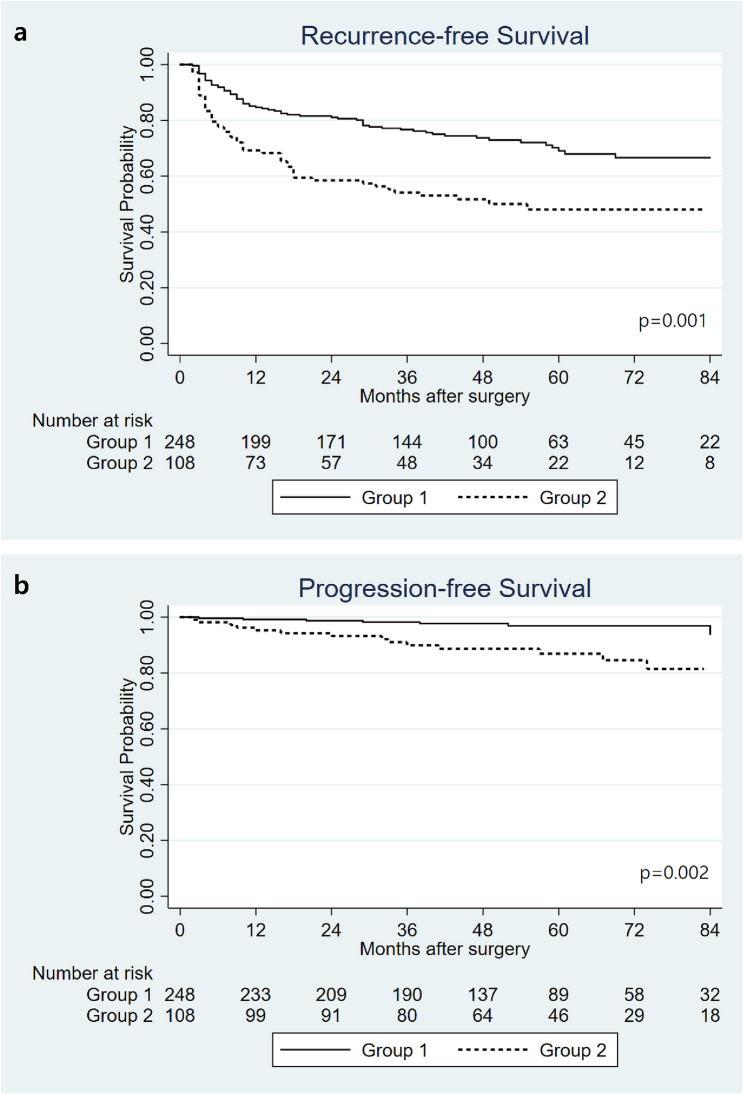
Kaplan–Meier curves for recurrence-free survival (**a**) and progression-free survival (**b**) in group 1 (prostate volume ≤ 30 mL) and group 2 (prostate volume > 30 mL) before propensity score matching.

In the median follow up duration of 52 months (interquartile range: 38–72 months), 66 patients (26.6%) experienced recurrence in group 1 and 53 patients (49.1%) in group 2, while 8 patients (3.2%) experienced progression in group 1 and 14 patients (13.0%) in group 2. Figure 1 showed Kaplan–Meier curves of recurrence-free survival (RFS) and progression-free survival (PFS) in two groups. The 5-year RFS was higher in group 1 than in group 2 (69.3% vs 47.0%, p = 0.001), and so was the PFS (96.7% vs 87.7%, p = 0.002).

Multivariable cox-regression analysis showed that tumor size (hazard ratio [HR] = 1.286, 95% confidential interval [CI] = 1.119–1.477, p < 0.001) and multifocal tumor occurrence (HR = 1.838, 95% CI = 1.251–2.700, p = 0.002) were associated with RFS. Adjuvant intravesical BCG instillation was associated with better RFS (HR = 0.605, 95% CI = 0.394–0.929, p = 0.022), although intravesical chemotherapy did not lower RFS (HR = 0.634, 95% CI = 0.372–1.081, p = 0.094). High grade of tumor (HR = 4.411, 95% CI = 14.379–14.11, p = 0.012) was associated with worse PFS, and high PV was a significant bad prognostic factor for both RFS (HR = 2.176, 95% CI = 1.508–3.141, p < 0.001) and PFS (HR = 3.213, 95% CI = 1.325–7.791, p = 0.010) (Table 2).

**Table 2:** Multivariable analyses of factors associated with recurrence-free survival and progression-free survival with all cohort.

Variables	Recurrence-free survival		Progression-free survival	
	HR (95% CI)	p-value	HR (95% CI)	p-value
Age	1.008 (0.993–1.023)	0.280	1.040 (0.998–1.085)	0.064
BMI	0.986 (0.924–1.054)	0.686	0.987 (0.839–1.162)	
**Smoking**		0.515		0.257
Non-smoker	1 (Ref.)		1 (Ref.)	
Current or former smoker	1.151 (0.753–1.760)		0.582 (0.229–1.482)	
Tumor size	1.286 (1.119–1.477)	< 0.001	0.891 (0.613–1.294)	0.544
Multifocal tumor	1.838 (1.251–2.700)	0.002	1.726 (0.648–4.600)	0.275
**Tumor grade**		0.585		0.012
Low grade	1 (Ref.)		1 (Ref.)	
High grade	0.889 (0.582–1.357)		4.411 (1.379–14.11)	
**Pathologic T stage**		0.071		0.555
pTa	1 (Ref.)		1 (Ref.)	
pT1	2.041 (0.940–4.429)		1.938 (0.216–17.41)	
CIS	2.362 (0.960–6.227)	0.082	1.828 (0.314–10.63)	0.502
Immediate intravesical therapy	0.811 (0.431–1.529)	0.518	0.756 (0.157–3.649)	0.728
**Adjuvant intravesical therapy**				
No	1 (Ref.)		1 (Ref.)	
Chemotherapy	0.634 (0.372–1.081)	0.094	0.152 (0.019–1.225)	0.077
BCG	0.605 (0.394–0.929)	0.022	0.845 (0.338–2.115)	0.720
**Prostate volume**		< 0.001		0.010
≤ 30 mL	1 (Ref.)		1 (Ref.)	
> 30 mL	2.176 (1.508–3.141)		3.213 (1.325–7.791)	

*HR* hazard ratio, *BMI* body mass index, *CIS* carcinoma in situ, *BCG* bacillus Calmette–Guérin.

### Recurrence-free and prognosis-free survival in the 2 groups (after PSM)

**Figure 2:**
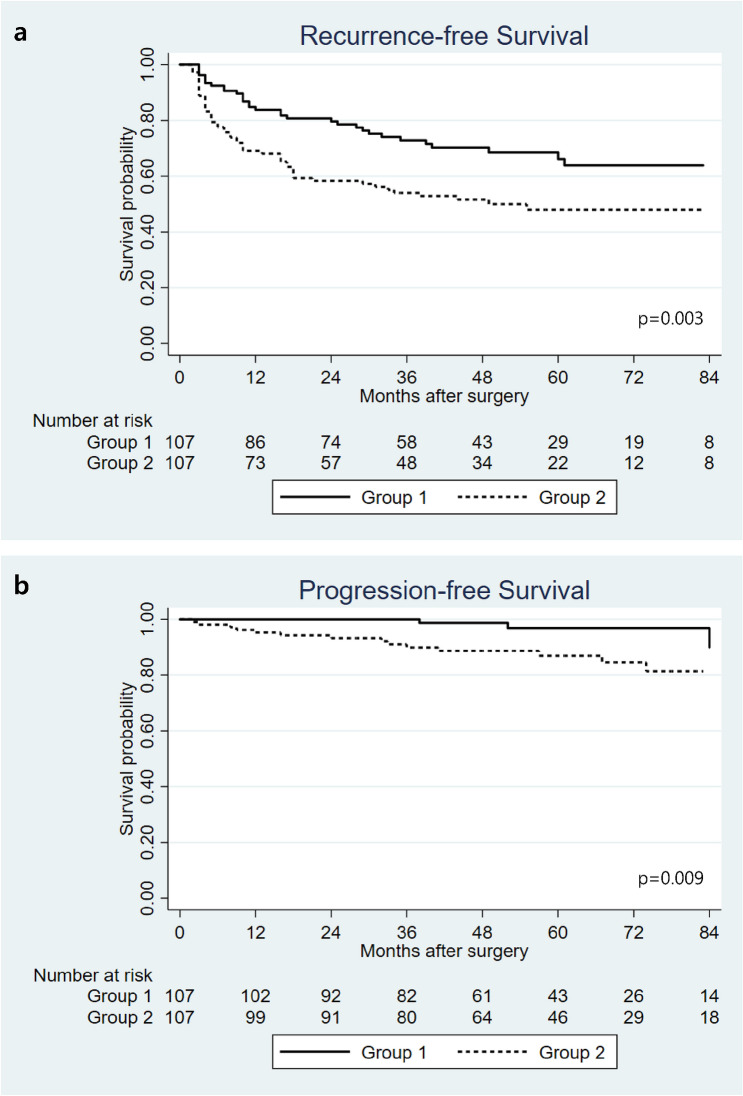
Kaplan–Meier curves for recurrence-free survival (**a**) and progression-free survival (**b**) in group 1 (prostate volume ≤ 30 mL) and group 2 (prostate volume > 30 mL) after propensity score matching.

Figure 2 showed Kaplan–Meier curves of RFS and PFS in matched cohort. The 5-year RFS was higher in group 1 than in group 2 (66.2% vs 47.9%, p = 0.003), and so was the PFS (96.9% vs 86.9%, p = 0.009).

Table 3 demonstrated multivariable cox-regression analysis after PSM and it showed similar results of pre-matched analysis. Tumor size (HR = 1.243, 95% CI = 1.050–1.470, p = 0.011) and multifocal tumor occurrence (HR = 1.787, 95% CI = 1.128–2.832, p = 0.013) were associated with RFS. Adjuvant intravesical BCG instillation was also associated with better RFS (HR = 0.575, 95% CI = 0.345–0.959, p = 0.034). High grade of tumor (HR = 5.939, 95% CI = 1.080–16.18, p = 0.040) was associated with worse PFS. High PV was a significant bad prognostic factor for both RFS (HR = 2.060, 95% CI = 1.324–3.206, p = 0.001) and PFS (HR = 4.855, 95% CI = 1.391–16.94, p = 0.013).

**Table 3:** Multivariable analyses of factors associated with recurrence-free survival and progression-free survival with propensity score matched cohort.

Variables	Recurrence-free survival		Progression-free survival	
	HR (95% CI)	p-value	HR (95% CI)	p-value
Age	1.005 (0.987–1.024)	0.555	1.012 (0.961–1.066)	0.641
BMI	0.986 (0.910–1.067)	0.724	0.981 (0.815–1.180)	0.835
**Smoking**		0.490		0.144
Non-smoker	1 (Ref.)		1 (Ref.)	
Current or former smoker	1.194 (0.721–1.978)		0.447 (0.152–1.317)	
Tumor size	1.243 (1.050–1.470)	0.011	0.864 (0.552–1.351)	0.521
Multifocal tumor	1.787 (1.128–2.832)	0.013	1.741 (0.527–5.755)	0.363
**Tumor grade**		0.292		0.040
Low grade	1 (Ref.)		1 (Ref.)	
High grade	0.760 (0.456–1.266)		5.939 (1.080–16.18)	
**Pathologic T stage**		0.359		0.729
pTa	1 (Ref.)		1 (Ref.)	
pT1	1.515 (0.623–3.683)		0.667 (0.068–6.574)	
CIS	2.030 (0.522–7.894)	0.307	1.601 (0.257–9.965)	0.614
Immediate intravesical therapy	0.519 (0.202–1.337)	0.174	1.348 (0.231–7.869)	0.74
**Adjuvant intravesical therapy**				
No	1 (Ref.)		1 (Ref.)	
Chemotherapy	0.624 (0.335–1.163)	0.138	0.191 (0.022–1.626)	0.130
BCG	0.575 (0.345–0.959)	0.034	1.158 )0.400–3.354)	0.787
**Prostate volume**		0.001		0.013
≤ 30 mL	1 (Ref.)		1 (Ref.)	
> 30 mL	2.060 (1.324/3.206)		4.855 (1.391–16.94)	

*HR* hazard ratio, *BMI* body mass index, *CIS* carcinoma in situ, *BCG* bacillus Calmette–Guérin.

## Discussion

There are various prognostic factors of NMIBC such as old age, female sex, presence of CIS, tumor size, tumor multifocality, tumor stage, and tumor grade. Some studies have shown that BPH is associated with an increased risk of bladder cancer; an epidemiological case–control study reported that previous prostatic surgery due to BPH was associated with increased risk of bladder cancer (relative risk = 2.38)^9^. A recent study reported that moderate to severe lower urinary tract symptoms (LUTSs) were associated with poor prognosis of NMIBC^16^. It has also been reported that the International Prostate Symptom Score was a significant predictor of recurrence of NMIBC (odds ratio = 1.26, p = 0.005). LUTSs are commonly associated with bladder outlet obstruction (BOO), which is often caused by benign prostatic enlargement^17^. Therefore, it is possible that the high PV is also related to the prognosis; hence, this study focused on PV.

We reviewed another study that gave a glimpse of the possible link between prostate and bladder cancer. Izumi et al. reported that the 5-year RFS was higher in the ADT group than in the control group (76% vs 40%, p < 0.001). They also showed that ADT was an independent prognostic factor for bladder cancer recurrence (HR = 0.29, p < 0.001)^12^. Other studies have reported that androgen suppression therapies such as 5-ARI and ADT reduce the risk of intravesical recurrence of bladder cancer (HR = 0.36, p = 0.024)^11^. The authors explain that the results of these studies could be related to androgen receptors (ARs). In fact, many studies have reported that AR signaling in bladder cancer cases was associated with recurrence and progression of bladder cancer^18,19^. Wang et al. also reported that 5-ARI might lower mortality related with bladder cancer^20^. Based on these results, we concur that recurrence of bladder cancer is more likely to be due to AR signaling. And previous studies have reported that AR plays an important role in enhancing cell growth in both stromal and epithelial cells, and it promotes development of BPH^15,21^. Referring to those studies, the size of the prostate may also have some influence on the prognosis of bladder cancer.

Although there are many modalities to measure PV, transrectal ultrasonography (TRUS) with the elliptical volume formula is most frequently used. However, we measured the PV using preoperative computed tomography (CT) performed for staging because TRUS was not available to all patients who had undergone TURBT. TRUS has clear advantages, including less radiation, ready availability, and cost-effectiveness^22^. On the other hand, it has the significant disadvantage of intra-operator variability. Zlotta et al.^23^ reported that the variability in the PV measurement ranges from -21% to 30%. Although some previous studies had reported PV was measured to be larger in CT than TRUS, more recent studies showed no differences^24^. We also found no significant difference in PV between TRUS and CT (28.8 mL vs 28.3 mL, p = 0.260) in 77 patients who received TRUS before surgery in our study.

Our study showed that high PV was associated with both recurrence and progression. RFS and PFS were higher in group 1 than in group 2. Multivariable cox analysis showed that a greater PV was associated with worse RFS and PFS. The hypothesis for the cause of these results is that, as mentioned above, the prognosis of bladder cancer is related to AR signaling. AR is likely to be relatively suppressed in people with small prostates, thereby suppressing the recurrence and progression of bladder cancer. Another possible explanation for the results is the effect of residual urine. 5-ARI reduces PV by the activity of androgen-regulated growth factor, which is ultimately responsible for angiogenesis. In fact, a previous study showed that the expression of VEGF was reduced in the prostate of patients with huge prostate using 5-ARI^25^. And decreased PV with using 5-ARI contributes to reducing residual urine^26^. In addition, a previous animal study, Kadlubar et al. reported that as the frequency of urination increased, the level of carcinogen in the urothelium decreased^27^. Another study showed that the higher the fluid intake, the lower the risk of bladder cancer^28^. These studies suggest that the risk and recurrence of bladder cancer increase with higher carcinogen exposure.

In addition to the pathological characteristics of bladder cancer, there have been various studies on factors affecting the prognosis of bladder cancer. Regarding various chronic diseases, Ferro et al.^29^ reported that type 2 diabetes mellitus increased the risk of recurrence and progression of high-grade MIBC. Regarding hematological characteristics, there was a study that reported baseline basophil might be predictor of BCG-treated high-grade NMIBC^30^. In addition, there have been studies that circulating tumor cells are also an important prognostic factor for bladder cancer^31,32^. For the prediction of recurrence and progression of NMIBC, the development of a predictive model that includes various factors including prostate size will help in counseling patients, making treatment decisions and making follow-up decisions.

Our study has some limitations. Although we established the relationship between PV and prognosis of NMIBC, the mechanism is still unknown. We believe that AR and residual urine play an important role. In order to confirm this in more depth, it would be helpful to assess the relationship between residual urine and the prognosis of NMIBC, including female population. Moreover, we have not been able to determine how therapeutic measures and transurethral resection of the prostate (TURP) for prostatic hyperplasia affect the prognosis of bladder cancer. Ham et al.^33^ reported that simultaneous TURP with TURBT reduces recurrence rate in men with BOO. They reported concomitant TURP was a significant factor in predicting recurrence of NMIBC without increased risk of recurrences in the bladder neck or prostatic urethra. However, subsequent studies did not increase the risk of recurrence of the bladder neck or prostatic urethra, but reported that it did not significantly affect the recurrence of the bladder. Randomized control study demonstrated concurrent TURP was associated with better RFS, although it was not statistically significant (HR = 0.294, p = 0.083)^34^. A study on how treatment of large PV affects the recurrence of bladder cancer in appropriate patient groups will help to better understand the relationship between prostate size and the prognosis of bladder cancer.

Another drawback is that our results might be sensitive to selection bias because this study was retrospective and non-randomized in nature. The number of patients included in this study was also relatively small, and the follow-up duration could have been longer. These could reason out why prognostic factors previously known to affect prognosis were found not to be associated. Only PV and tumor grade were especially associated with progression of NMIBC.

Despite of those drawbacks, our study showed that PV is an independent prognostic factor of NMIBC. High PV was found to be associated with worse RFS and PFS. Our study suggested that PV reduction could be helpful for prevention of recurrence and progression in patients with NMIBC.

## Methods

### Patient selection

**Figure 3:**
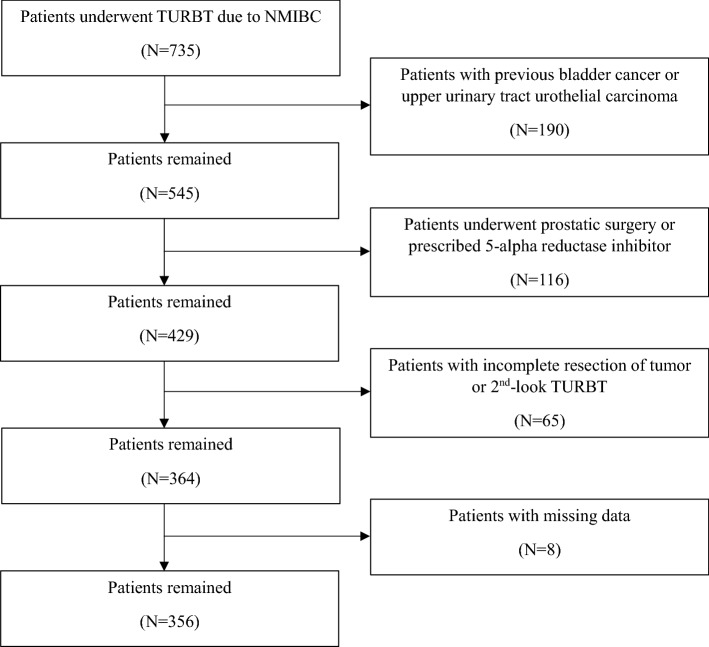
Flowchart of patient selection.

We reviewed the medical records of 735 men who underwent TURBT due to NMIBC without regional lymph node or distant metastasis between January 2012 and December 2017 at Severance Hospital, Seoul, Korea. The exclusion criteria were as follows: (1) patients who had undergone previous TURBT; (2) patients who had undergone surgery for upper urinary tract urothelial carcinoma; (3) patients who had undergone previous or concurrent prostatic surgery, such as TURP or prostatectomy; (4) patients who were prescribed with 5-ARI; (5) patients with incomplete resection of tumor or second-look TURBT; and (6) patients with missing data and short follow-up duration. Finally, 356 patients were included in this study (Fig. 3).

### Patient characteristics

We investigated the following data in all patients: age, BMI, tumor size and multifocality, tumor grade, pathologic tumor stage, presence of CIS, intravesical therapy and PV. PV was assessed using a CT scan, which was performed for the purpose of staging before TURBT, and was calculated using the ellipsoid formula referring to a previous study: 0.52 × [width (cm)] × [length (cm)] × [height (cm)]^24^. The assessment was performed twice by two urologists, and the average value was determined.

Patients were divided into two groups based on their calculated PV: group 1 included 248 patients with PV ≤ 30 mL, and group 2 included 108 patients with PV > 30 mL. These two groups were compared for the previously mentioned variables to evaluate whether there was a statistically significant difference between them.

### Follow-up

The follow-up schedule including intravesical BCG therapy or chemotherapy, which decided by the attending physician based on the classification of cancer risk. Cystoscopy with urine cytology was performed every 3 months for the first year, every 6 months for the second year, and every 6–12 months thereafter. Evaluation for upper urinary tract or distant metastasis was performed every 6–12 months, as needed. Tumor recurrence was defined with confirmation on histopathological examination when it was suspected on cystoscopy. Tumor progression was defined as a diagnosis of muscle invasive bladder cancer, or regional node or distant metastasis.

### Statistical analyses

Patient characteristics were compared between the two groups using the Mann–Whitney *U* test for continuous data and Chi-squared test for dichotomous variables. Categorical variables are presented as frequencies and percentages, whereas continuous variables are expressed as medians and interquartile ranges. Then propensity score matching was performed using five factors which showed statically difference between two groups: age, BMI, tumor size, tumor grade and pathologic T stage (Caliper: 0.01). With propensity-score matched cohort, Kaplan–Meier curves and the log-rank test were used to depict and compare RFS and PFS between the two groups. Multivariable Cox regression models were constructed to determine the variables associated with RFS and PFS. All tests were two-sided, with statistical significance considered at p < 0.05. The statistical analyses were performed using STATA^®^ version 15.1 (StataCorp LLC, College Station, TX, USA).

### Good clinical practice protocols

The study protocol was approved by the institutional review board of the Yongin Severance Hospital. (Approval number: 9-2020-0173) And this study was performed in agreement with the applicable laws and regulations, good clinical practices, and ethical principles described in the Declaration of Helsinki s and written informed consent was obtained from all patients.
